# Characterization of microbial communities in heavy crude oil from Saudi Arabia

**DOI:** 10.1007/s13213-014-0840-0

**Published:** 2014-03-12

**Authors:** Majed Albokari, Ibrahim Mashhour, Mohammed Alshehri, Chris Boothman, Mousa Al-Enezi

**Affiliations:** 1 Atomic Energy Research Institute (AERI), King Abdulaziz City for Science and Technology (KACST), P. O. Box 6086, Riyadh, 11442 Saudi Arabia; 2 School of Earth, Atmospheric and Environmental Sciences and Williamson Research Centre for Molecular Environmental Science, University of Manchester, Manchester, M13 9PL UK; 3 Saudi Aramco, Research & Development Center, P.O. Box 62, Dhahran, 31311 Saudi Arabia

**Keywords:** Heavy crude oil, Oil sludge, 16S rRNA, PCR amplification, Saudi Aramco oil company

## Abstract

The complete mineralization of crude oil into carbon dioxide, water, inorganic compounds and cellular constituents can be carried out as part of a bioremediation strategy. This involves the transformation of complex organic contaminants into simpler organic compounds by microbial communities, mainly bacteria. A crude oil sample and an oil sludge sample were obtained from Saudi ARAMCO Oil Company and investigated to identify the microbial communities present using PCR-based culture-independent techniques. In total, analysis of 177 clones yielded 30 distinct bacterial sequences. Clone library analysis of the oil sample was found to contain *Bacillus*, *Clostridia* and *Gammaproteobacteria* species while the sludge sample revealed the presence of members of the *Alphaproteobacteria*, *Betaproteobacteria*, *Gammaproteobacteria*, *Clostridia*, *Spingobacteria* and *Flavobacteria*. The dominant bacterial class identified in oil and sludge samples was found to be *Bacilli* and *Flavobacteria*, respectively. Phylogenetic analysis showed that the dominant bacterium in the oil sample has the closest sequence identity to *Enterococcus aquimarinus* and the dominant bacterium in the sludge sample is most closely related to the uncultured *Bacteroidetes* bacterium designated AH.KK.

## Introduction

Huge amounts of organic substances principally exist in crude oil and oil sludge as hydrocarbons (HCs such as polyaromatic, alicyclic, etc.) along with a range of different mixtures of oxygen, nitrogen, sulphur-containing organic compounds and inorganic matter (such as metals, sulfides and thiophenes). Oil occurs in sedimentary layers, often in association with water, which can make up between 5 % and 20 % of the sedimentary layer; this percentage generates high levels of salts. The presence of macroelements and microelements in crude oil depends on the surrounding environment and the nature of the crude oil’s bed (Yemashova et al. 2007).

Contamination of different environments by oil (soil, landscape, aquatic, etc.) associated with oil activities (exploration, production, transferring, etc.) are a major concern. For example, oil spills occur due to leakage from pipelines, accidents involving oil tankers during transportation, and anthropogenic and pilferage activities. Oil sludge contamination contains hazardous hydrocarbons, which is also a major concern. The term “oil sludge” designates waste generated due to storage of crude/products, containing mixtures of oil, water and solids. Since many of its constituents are highly toxic, carcinogenic and are poorly degradable in nature (Sarkar et al. 2005; Das and Chandran 2011), treating oil-contaminated sites/environments using chemical reagents, which is one of the conventional methods, is not preferable for a number of reasons, one of which is the high costs involved. These conventional methods can be replaced by modern bioremediation strategies involving the stimulation of indigenous microorganisms, or the addition of modified/engineered biota, that have the ability to degrade such hydrocarbon compounds to either small simple nontoxic molecules (carbon dioxide and water) or biomass under aerobic conditions (Philip et al. 2008). Various groups of microorganisms have been identified that are able to degrade petroleum hydrocarbons by utilizing the available hydrocarbons as sole sources of carbon and energy (Jain and Bajpai 2012).

The natural process of biodegradation can be sped up if selective and effective nutrients are added to the eco/environmental systems, this will promote the growth of microorganisms capable of carrying out remediation activities (Khan and Rizvi 2011). For example, microbiologically influenced corrosion is the term used for the phenomenon in which corrosion is initiated, accelerated or both, by the activities of microorganisms in buried pipelines. This is the case in crude oil pipelines, which are subject to microbiologically influenced corrosion, particularly in water pockets at low-lying sections of the pipeline (Al-Saleh et al. 2011). A recent review prepared by Philip et al. (2008) summarized different biotechnological processes from a large number of research articles based on petroleum microbiology, and dedicated to increasing the knowledge of the bacteriology of petroleum reservoirs. However, The Environmental Protection Agency (U.S. EPA 2008) has reviewed the available and common remediation technologies with their associated costs and bioremediation processes were amongst the best candidates.

Linking the characterization and quantification of microbial communities with remediation processes involving specific functional groups can be technically challenging, especially in oil-contaminated samples. In addition, the composition of oil-contaminated sites may be harmful to most biota (flora), leading to a reduction in their population. On the other hand, the presence of both energy sources represented by carbon substrates, and subsequent metabolites in oil-contaminated sites, increases the possibility of the formation and development of complex microbial communities (Van Hamme et al. 2003).

Microbial communities in crude oil from different locations worldwide like Japan, China and South Africa have been identified and characterized by different methods and techniques. The 16S rRNA gene-based clone libraries for samples from two platforms off shore in Brazil were dominated by members of the *Gammaproteobacteria*, with a smaller number of clones associated to *Betaproteobacteria*, *Alphaproteobacteria* and *Firmicutes* (Korenblum et al. 2012). *Gammaproteobacteria* were mainly represented by *Pseudomonadaceae* along with several species of *Moraxellaceae*, *Enterobacteriaceae*, *Alteromonadaceae* and *Xanthomonadaceae*. The *Betaproteobacteria* present belonged to the *Comamonadaceae*, *Burkholderiaceae* and *Oxalobacteraceae* while the *Alphaproteobacteria* were from the SAR11 clade. The *Firmicutes* were reported as *Bacillaceae* and *Veillonellaceae* from the study by Korenblum et al. (2012). Moreover, similar bacterial communities were obtained by Liu et al. (2009) from crude oil samples in the Changqing Oil field in China and by Yoshida et al. (2005) in crude-oil samples from Japanese oil stockpiles. In both studies, bacteria related to *Ochrobactrum* sp., *Stenotrophomonas* sp., *Burkholderia* sp., *Brevundimonas* sp. and *Propionibacterium* sp. were detected in the crude oil samples (Yoshida et al. 2005; Liu et al. 2009). In the local region of the Arabian Gulf, where the largest crude oil reservoir exists, several crude oil samples were collected from seven locations along the coast of Kuwait. Here, using 16S rRNA analysis, the bacterial communities were found to be dominated by several genera, mainly *Pseudomonas*, *Bacillus*, *Staphylococcus*, *Acinetobacter*, *Kocuria* and *Micrococcus* (Al-Saleh et al. 2009). Abundant microbial communities from a crude oil sludge environment (Qua Iboe Terminal (QIT), Eket, Nigeria) were identified as *Micrococcus varians*, *Bacillus subtilis* and *Pseudomonas aeruginosa* (Ekpo and Udofia 2008). The study was designed to monitor the rate of the biodegradation of hydrocarbons in soil by microorganisms isolated from a crude oil sludge environment. The above five studies demonstrate that the documentation of the bacterial community diversity within each site is of the utmost importance. This is because the documentation work includes isolation, identification and characterization that will lead to the discovery of novel bacterial strains, which have the capability to degrade and clean-up intractable complex compounds (Jain and Bajpai 2012). In Saudi Arabia, relatively little work has been carried out on studying microbial communities. However, examples of studies in this area include that made by Al-Thukair et al. (2007) on cyanobacterial mats in the intertidal zone of the oil-polluted coast of the Arabian Gulf. Raeid et al. (2006) have also studied the bacterial diversity of cyanobacterial mats which have the ability to degrade petroleum compounds from the Arabian Gulf coast of Saudi Arabia (Raeid et al. 2006; Al-Thukair et al. 2007), Other studies focused on the isolation of selective bacterial species inhabiting crude oil and petroleum contaminated areas for utilization in biotechnological applications such as Bioremediation and Bioaugmentation (Binsadiq 2013). Therefore, the work presented here aims to characterize the microbial communities of the oil and oil sludge samples, associated with exploration and production of heavy crude oil from Saudi Arabia by the Saudi ARAMCO Company. Attempts were also made to compare these bacteria with those found in other worldwide oil reservoirs, and illustrate the potential bacteria that can be utilized further for bioremediation.

## Materials and methods

### Materials

The samples were collected and delivered by the technical team of Saudi Aramco Oil Company due to access restrictions to the oil field sites. Oil and sludge samples were collected from the Eastern province of Saudi Arabia, where most of the oil fields are located. The oil sample is considered a heavy type taken directly from the refinery and the sludge sample was taken from the nearby lagoon.

### Methods

The Powersoil DNA Isolation kit was used for the extraction of DNA from oil and oil sludge samples. The DNA extraction from the oil sample was perfumed in triplicate and pooled due to the low level of biomass present within the sample.

### DNA extraction and amplification of 16S rRNA gene sequences

DNA was extracted from oil (0.6 g) and sludge (0.2 g) samples using a PowerSoil DNA Isolation Kit (PowerSoil DNA Isolation Kit, MO BIO Laboratories INC, Solana Beach, CA, USA). Almost the whole gene (1541 in *E. coli*) of 16S rRNA, approximately 1,490 bp, was amplified from samples using the broad-specificity primers 8F (Eden et al. 1991) and 1492R (Lane et al. 1985). PCR reactions were performed in thin-walled tubes using a BioRad iCycler (BioRad, Hemel Hempstead, Herts, UK). Takara Ex Taq Polymerase (Millipore U.K LTD, Watford, UK) was used to amplify DNA from the sample extract. The PCR amplification protocol used with the 8F and 1492R primers was: initial denaturation at 94 °C for 4 min, melting at 94 °C for 30 s, annealing at 50 °C for 30 s, elongation at 72 °C for 3 min; 35 cycles, followed by a final extension step at 72 °C for 5 min. Purity of the amplified products was determined by electrophoresis in Tris-acetate-EDTA (TAE) gel. DNA was stained with ethidium bromide and viewed under short wave UV light using a BioRad Geldoc 2000 system (BioRad, Hemel Hempstead, Herts, UK).

The resulting PCR products were purified using an ExoSap protocol, 2 μl of ExoSap mix (0.058 μl Exonuclease I, 0.5 μl Shrimp Alkaline Phosphatase and 1.442 μl QH_2_O) was added to 5 μl of PCR product and incubated at 37 °C for 30 min followed by 80 °C for 15 min. Purified gene fragments were ligated directly into a cloning vector using a PCR cloning kit (StrataClone, Stockport, UK) containing topoisomerase I-charged vector arms (Agilent Technologies, Wokingham, UK) prior to transformation into *E. coli* competent cells expressing Cre recombinase (Agilent Technologies, Wokingham, UK). Positive clones (96 per library) were screened by PCR using primers complementary to the flanking regions of the PCR insertion site of the cloning vector, and sequenced using the ABI Prism® BigDye™ Terminator v3.1 Cycle Sequencing Kit (Applied Biosystems, Life Technologies Corporation, USA). The forward primer, 8F (Eden et al. 1991) was used for the sequencing reaction.

**Table 1:** Closest cultured strains of bacteria based on 16S rRNA Gene libraries from the sludge sample

Class	Closest match	% Clone library	% Match	Accession No.
*Flavobacteria*	Uncul. *Bacteroidetes* bacterium AH.KK.	54.4	(98.4)	GQ979644
		54.4		
*Sphingobacteria*	Uncul. *Bacteroidetes* bacterium; AL07-21	12.2	(90.3)	FJ843776
		11.1		
	*Gracilimonas tropica*	1.1	(96.2)	EF988655
*Gammaproteobacteria*	*Marinobacter gudaonensis*	27.5	(97.7)	DQ414419
		5.6		
	Uncultured *Gammaproteobacterium* clone bac463	4.4	(98.3)	JF727683
	*Idiomarina loihiensis*	4.4	(99.9)	AF288370
	*Marinobacter pelagius*	3.3	(97.4)	DQ458821
	Uncultured bacterium; *Napoli-*MN16BT2-230	2.2	(98.6)	AY593468
	*Marinobacter lipolyticus*	1.1	(99.6)	AY147906
	*Chromohalobacter* sp. H24.1	1.1	(100)	AJ717726
	*Hydrocarboniphaga effusa*	1.1	(94.8)	AY363245
	*Halomonas ventosae*	1.1	(99.9)	AY268080
	*Marinobacter guineae*	1.1	(98.5)	AM503093
	*Chromohalobacter salexigens*; SM5	1.1	(97)	HQ641752
*Betaproteobacteria*	Uncultured *Alcaligenes* sp.; Y251	1.1	(99.3)	EU328096
		1.1		
*Alphaproteobacteria*	*Thalassospira lucentensis*; TVGB18	4.4	(99.5)	GQ169074
		2.2		
	*Parvibaculum lavamentivorans*	1.1	(97)	AY387398
	Uncultured *Parvibaculum* sp.; HB83	1.1	(97.5)	EF648080
*Clostridia*	*Halanaerobium acetethylicum*	1.1	(99.8)	X89071
		1.1

**Table 2:** Closest cultured strains of bacteria based on 16S rRNA Gene libraries from oil sample

Class	Closest Match	% Clone Library	% Match	Accession No.
*Bacilli*	*Enterococcus aquimarinus* (T); LMG 16607	64.4	(97.4)	AJ877015
		55.3		
	*Enterococcus aquimarinus*; SS1788	4.6	(97.4)	GQ337015
	*Enterococcus inusitatus*; type strain: E2ET31	2.3	(95.3)	AM050563
	*Staphylococcus* sp. R-25050	1.1	(99.5)	AM084016
	*Amphibacillus* sp. DA-6	1.1	(98.1)	AB533308
*Clostridia*	*Clostridium* sp. enrichment culture clone MB3_7	34.5	(97)	AM933653
		19.6		
	Uncultured *Clostridium* sp. clone De155	11.6	(99)	HQ183771
	*Clostridium bifermentans*; DPH-1	1.1	(99.1)	EU526032
	*Clostridia* bacterium S130(5)-2	1.1	(99.3)	GU136559
	*Clostridium* sp. CYP7	1.1	(99)	DQ479417
*Gammaproteobacteria*	*Serratia fonticola*	1.1	(97.9)	AJ233429
		1.1		

### DNA sequencing and phylogenetic analysis

Nucleotide sequences were determined by the dideoxynucleotide method. An ABI Prism BigDye Terminator Cycle Sequencing Kit was used in combination with an ABI Prism 877 Integrated Thermal Cycler and ABI Prism 377 DNA Sequencer (Perkin Elmer Applied Biosystems, Warrington, UK). Sequences (typically 900 base pairs in length) were analysed using Mallard (Ashelford et al. 2006) to check for presence of chimeras or sequencing anomalies. Operational taxonomic units (OTU) were determined at a 98 % sequence similarity level using Mothur (Schloss et al. 2009). The individual OTU sequences were analysed using the sequencing database of known 16S rRNA gene sequences provided on the Ribosomal Database Project (Cole et al. 2009) to identify nearest neighbours. The sequences obtained were submitted to a BLAST search to retrieve the corresponding phylogenetic relatives. The phylogenetic affiliations were confirmed by analyses of all related species recognized by the taxonomic and classification hierarchy done with the NCBI Taxonomy database.

Three neighbour-joining phylogenetic trees were constructed to analyze the relationships among the sequences of the ribosomal library and related organisms from the GenBank database. The phylogenetic analysis were done using MEGA5.10 software (Tamura et al. 2011).

## Results

### Microbial community analysis

**Fig. 1:**
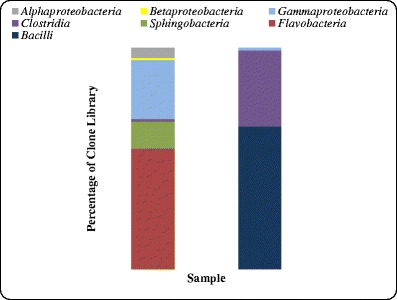
Phylogenetic Classes detected within the sludge (*left*) and oil (*right*) samples

**Fig. 2:**
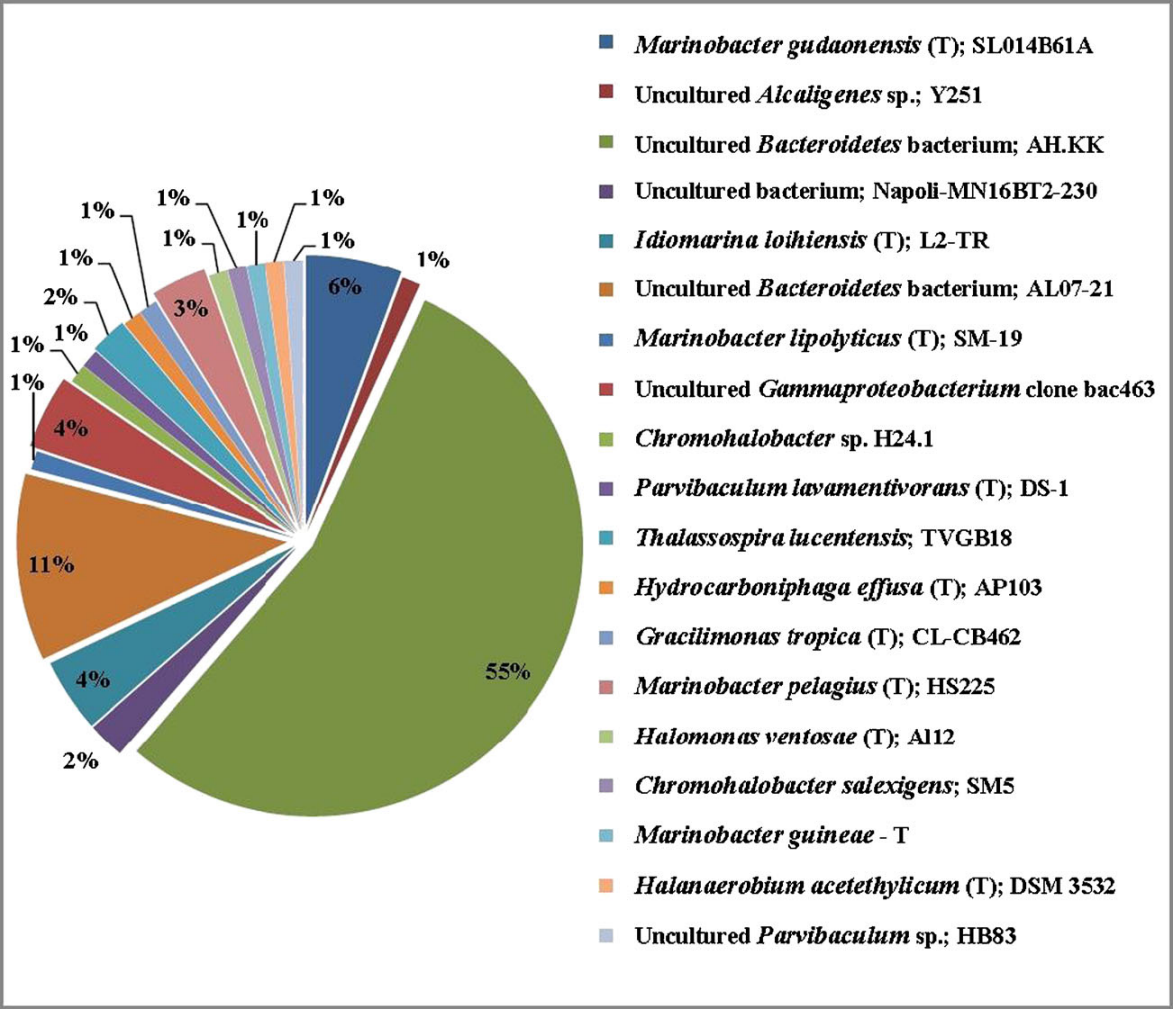
Closest Matching Microorganisms from 16S rRNA Analysis of the sludge sample

**Fig. 3:**
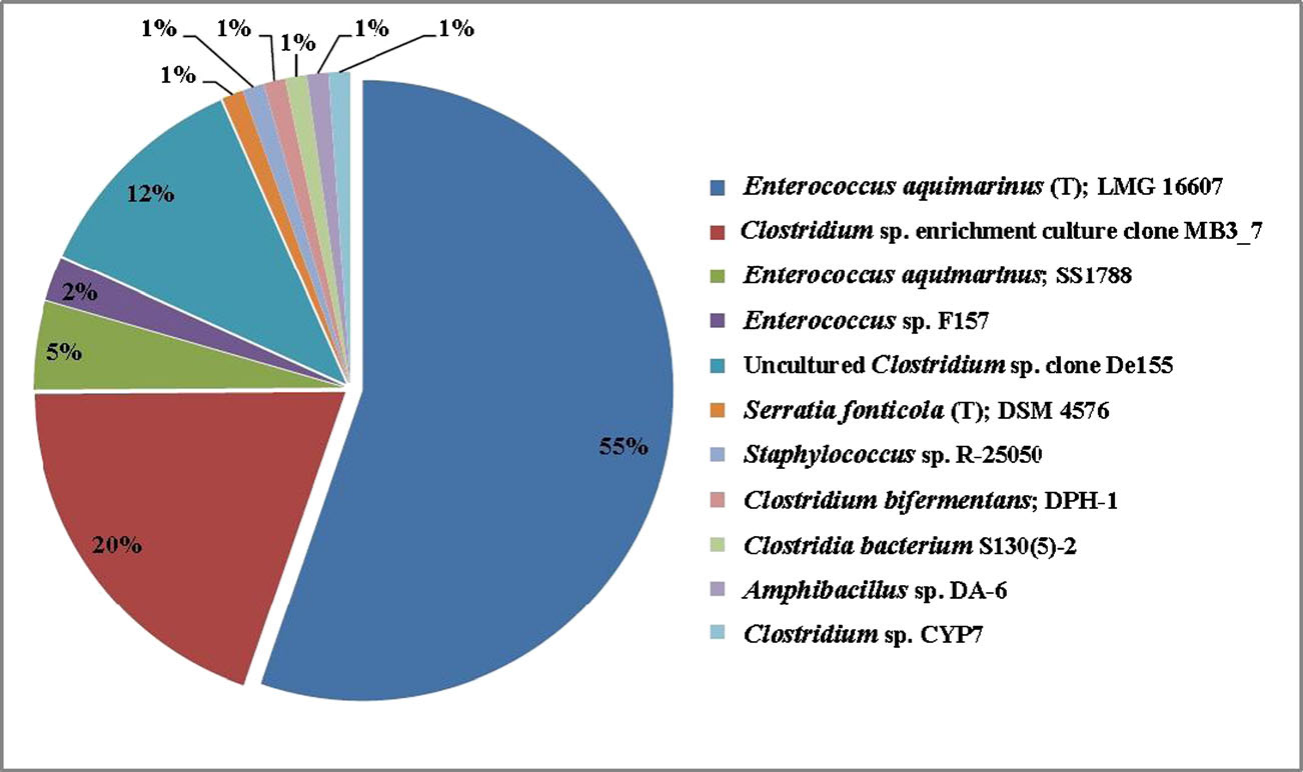
Closest Matching Microorganisms from 16S rRNA Analysis of the oil sample

In this study, two clone libraries were constructed from amplified community 16S rRNA genes. In total 177 clones (90 from sludge and 87 from oil) were obtained (Tables 1 and 2). Figure 1 summarizes the taxonomic compositions at the class level for each sample, demonstrating that the dominant phylogenetic class present in the sludge sample was *Flavobacteria*, comprising 54.4 % of the clone library. Additionally, this community was found to contain members of the *Alphaproteobacteria*, *Betaproteobacteria*, *Gammaproteobacteria*, *Spingobacteria* and *Clostridia. Bacilli* dominated the oil sample, making up 64.3 % of that clone library, the rest of this library comprised members of the *Gammaproteobacteria* and *Clostridia*. Closest cultured strains of bacteria based on 16S rRNA Gene libraries from oil sludge and oil samples are presented in Figs. 2 and 3, respectively.

A total of 90 clones were analysed from sludge sample, and results are presented in Fig. 2. The 16S rRNA gene sequences were tentatively assigned to the following types in the sludge sample: *Proteobacteria* (*Alpha*, *Beta* and *Gamma*), *Flavobacteria*, *Spingobacteria* and *Clostridia. Flavobacteria* were the most dominant group (54.4 % of the clone library); whose closest match was the uncultured *Bacteroidetes* bacterium AH.KK. The second most abundant phylogenetic class of bacteria detected were *Gammaproteobacteria* (5.6 % of the clone library), from which the closest matching species in the blast database was *Marinobacter gudaonensis*.

A total of 87 clones were analysed from the oil sample, and the results are presented in Fig. 3. The 16S rRNA gene sequences were tentatively assigned as *Bacilli*, *Clostridia* and *Gammaproteobacteria*, the *Bacilli* being the most dominant, from which the closest matching organism was *Enterococcus aquimarinus*.

**Fig. 4:**
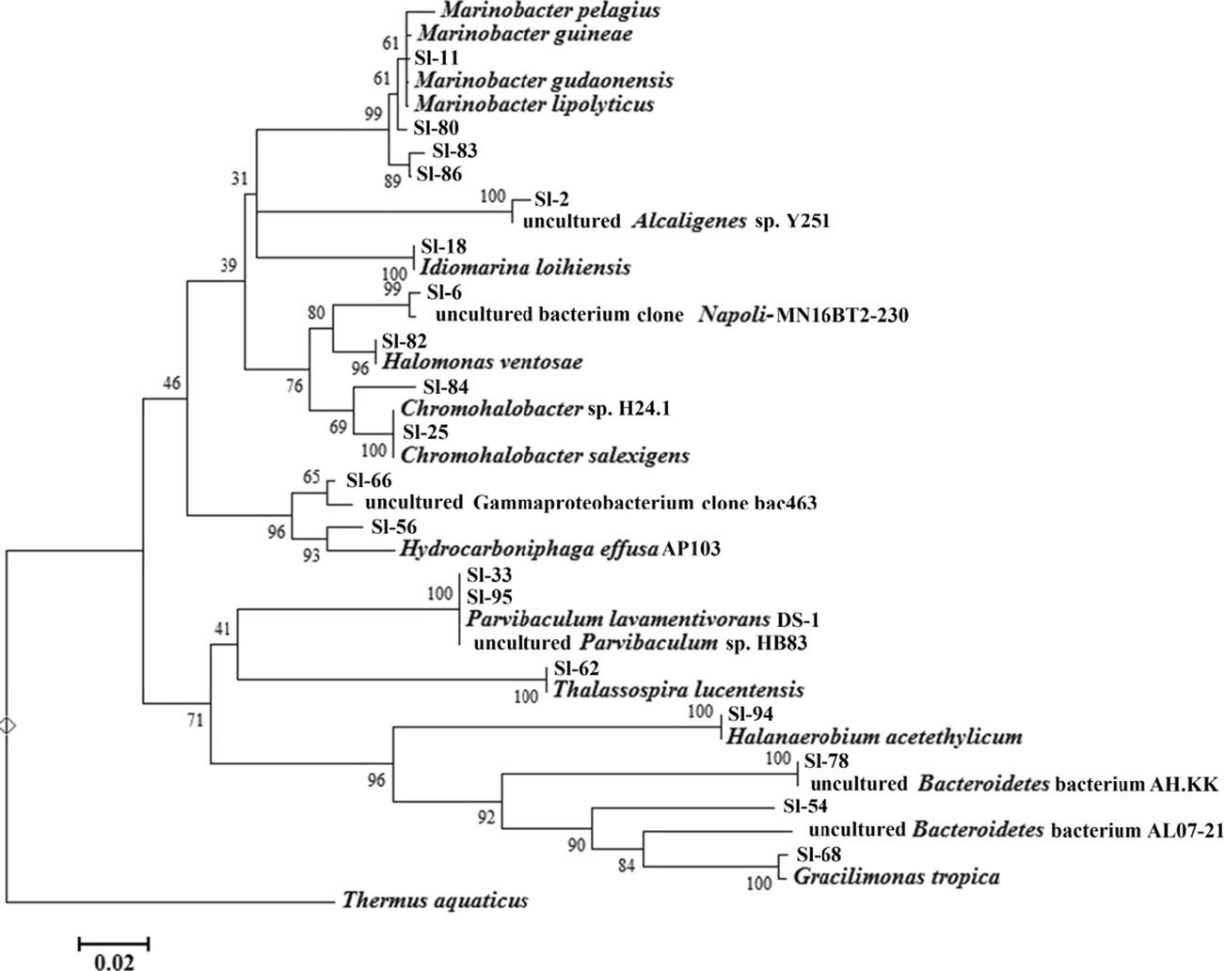
Bootstrap consensus tree inferred from 500 replicates, showing the closest matching microorganisms present in the sludge sample and their nearest relatives in the Blast database. *Scale bar* represents substitutions per site

**Fig. 5:**
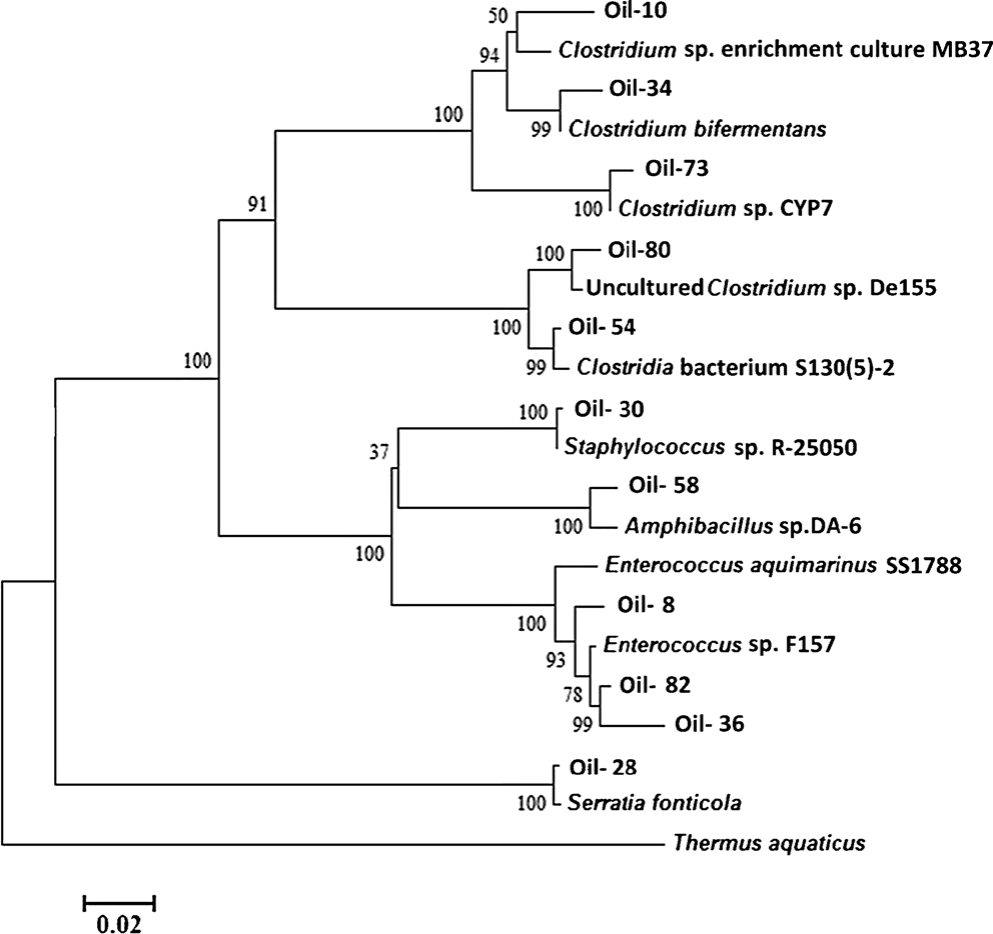
Bootstrap consensus tree inferred from 500 replicates, showing the closest matching microorganisms found within the oil sample and their nearest relatives in the Blast Database. *Scale bar* represents substitutions per site

Evolutionary history was inferred from both samples using the Neighbour-Joining method. Both bootstrap consensus trees were inferred from 500 replicates and are taken to represent the evolutionary history of the taxa analysed (Figs. 4 and 5). Branches corresponding to partitions reproduced in less than 50 % bootstrap replicates are collapsed. The percentage of replicate trees in which the associated taxa clustered together in the bootstrap test (500 replicates) are shown next to the branches. The tree is drawn to scale, with branch lengths in the same units as those of the evolutionary distances used to infer the phylogenetic tree. The evolutionary distances were computed using the Maximum Composite Likelihood method and are in the units of the number of base substitutions per site. The analysis involved 22 nucleotide sequences (oil tree) and 39 nucleotide sequences (sludge tree). Codon positions included were 1st+2nd+3rd+Noncoding. All positions containing gaps and missing data were eliminated. Evolutionary analyses were conducted in MEGA5.

## Discussion

Microbially influenced corrosion of crude oil pipelines has been a major cause of intermittent operational problems, and the frequency and severity of this problem has been increasing (Muthukumar et al. 2003). Microbial activity can result in inhibitor/fuel degradation that leads to problems including unacceptable level of turbidity, filter plugging, corrosion of storage tanks and pipelines and souring of stored products (Rajasekar et al. 2010). Inability to cultivate native bacteria within crude oil remains the main limitation for their exploitation. A PCR-based approach offers an alternative to microorganism isolation and cell counts, as a strategy to study the microbial diversity in order to gain an understanding of the uncultured microbial community (Korenblum et al. 2012). Therefore, microbial functional gene diversity in oil and sludge samples were analyzed in this study, by DNA extraction, the amplification of 16S rRNA gene sequences, followed by DNA sequencing and phylogenetic analysis.

The dominant bacterial genera identified were *Flavobacterium* and *Bacillus* in sludge and oil samples, respectively. However, dominant bacteria affiliated with genera *Phormidium*, *Schizothrix* and *Microcoleus* were identified in both studies produced from the Arabian Gulf, along with less dominant species belonging to *Spirochetes*, *Beta*-, *Gamma*- and *Deltaproteobacteria*, *Cytophaga*-*Flavobacterium*-*Bacteroides* group (Raeid et al. 2006; Al-Thukair et al. 2007). The dominant bacterial species identified in the petroleum pipeline of the northwest and southeast regions of India were *Bacillus cereus* and *Serratia marcescens* (Rajasekar et al. 2010). Representatives of the genus *Pseudomonas* were isolated from crude oil contaminated soil from Mersin, Turkey (Coral and Karagöz 2005). *Alpha* and *Gammaproteobacteria* were found to be abundant in oil polluted microbial mats from the Etang de Berre (France) (Hernandez-Raquet et al. 2006). Members of the *Pseudomonas*, *Thauera*, *Hydrogenophaga*, *Rhodoferax* and *Acidovorax* were found to be the dominant bacterial species in the sludge of an Alberta oil sands tailings pond (Golby et al. 2012). The study carried out by Jiménez et al. (Jiménez et al. 2007) investigated samples from the Prestige heavy fuel-oil spill on a beach on the Cantabrian coast (north Spain). Here the bacterial community structure was determined by cultivation-independent analysis of polymerase chain reaction-amplified 16S rDNA by denaturing gradient gel electrophoresis. The bacterial community was composed mainly of *Alphaproteobacteria* (*Rhodobacteriaceae* and *Sphingomonadaceae*). Representatives of *Gammaproteobacteria* (*Chromatiales*, *Moraxellaceae* and *Halomonadaceae*), *Bacteroidetes* (*Flavobacteriaceae*) and members of the *Actinobacteria* group (*Nocardiaceae* and *Corynebacteriaceae*) were also found to be presented. Within Brazilian petroleum reservoirs (de Oliveira et al. 2008) *Bacillus* sp. and *Halanaerobium* sp. were shown to be the predominant components of the bacterial community from the formation water sample, whereas the oil sample also included *Alicyclobacillus acidoterrestris*, *Rhodococcus* sp., *Streptomyces* sp. and *Acidithiobacillus ferrooxidans*. Crude oil samples with high-water and low-water content from two offshore platforms (PA and PB) in the Campos Basin, Brazil, were assessed for bacterial communities by 16S rRNA gene-based clone libraries. Here, the Ribosomal Database Project (RDP) Classifier was used to analyze a total of 156 clones within four libraries obtained from two platforms. The clone sequences were mainly affiliated with *Gammaproteobacteria* (78.2 % of the total clones); however, clones associated with *Betaproteobacteria* (10.9 %), *Alphaproteobacteria* (9 %) and *Firmicutes* (1.9 %) were also identified. *Pseudomonadaceae* was the most common family affiliated with these clone sequences (Korenblum et al. 2012).

The roles of *Bacillus* sp. in hydrocarbon bioremediation have been reported previously (Ijah and Antai 2003; Sepahi et al. 2008). Members of the phylum *Proteobacteria* are believed to be involved in the removal of organic pollutants, such as nitrogen, phosphorus and aromatic compounds. This suggests that some specific genera frequently detected in our samples are highly correlated to bacteria capable of carrying out the degradation of oil and sludge. The results of this study suggest that a large number of novel species may inhabit complex oil and sludge communities, and that they may possess qualities suited to their exploitation in full-scale bioremediation of oil contaminated sites.

Three bacterial isolates (*Flavobacterium* sp., *Acinetobacterium calcoaceticum* and *P. aeruginosa*) capable of utilizing used engine-oil as a carbon source were isolated from contaminated soils and identified using biochemical tests and 16S rRNA sequencing (Mandri and Lin 2007). Oil field bacteria present in different western Canadian oil fields were also characterized by cloning and sequencing of PCR-amplified 16S rRNA genes. In this case a variety of Gram-negative, sulfate-reducing bacteria of the family *Desulfovibrionaceae* and *Desulfobacteriaceae* were detected. In contrast, a much more limited number of anaerobic, fermentative, or acetogenic bacteria, *Clostridium* sp., *Eubacterium* sp. and *Synergistes* sp. were found. Potential sulfide oxidizers and/or microaerophiles (*Thiomicrospira*, *Arcobacter*, *Campylobacter* and *Oceanospirillum* sp.) were also detected (Voordouw et al. 1996).

In our study, a total of 90 clones were analyzed from the sludge sample. The dominant species present (∼54 % of the clone library) was found to be ∼98 % matched to uncultured *Bacteroidetes* bacterium AH.KK. In general, *Bacteroidetes* are Gram negative, chemolithotrophic, anaerobic bacteria that are widely distributed in nature. The phylum contains Fe(III)-reducing bacteria (Wang et al. 2010) and some organisms capable of carrying out oxidation of various sulfur species (Green et al. 2011). The addition of ferrihydrates with or without sulfate reduction inhibitor sodium molybdate may promote growth of these species, thus, enhancing biodegradation of crude oil. This isolate has previously been found in the unamended surface sediments of a tidal channel in the Tijuana River Estuary, California, USA as part of a study into the influence of microbial reducible Fe (III) (Cummings et al. 2010). In addition, *Marinobacter* species including *gudaonensis*, *lipolyticus*, *pelagius* and *guineae* were identified, comprising 5.6 %, 1.1 %, 3.3 % and 1.1 %, respectively, of the 16S clone library. Originally, *M. gudaonensis* was isolated from an oil-polluted saline soil from Gudao in the coastal Shengli Oilfield, eastern China (Gu et al. 2007) while *M. lipolyticus* was isolated from the hypersaline habitats of southern Spain (Martín et al. 2003). The latter is a halophilic bacterium exhibiting lipolytic activity which is promising for use in a variety of industrial applications. Similar to *M. lipolyticus*, *M. pelagius* and *M. guineae* are also considered halophilic bacterium (Montes et al. 2008, Xu et al. 2008). Two promising isolates, each contributing 1.1 % towards the 16S clone library of the oil sludge were recorded, *Hydrocarboniphaga effuse* and *Chromohalobacter* sp. H24.1. The former is an active bacterium in alkane and aromatic hydrocarbon degradation (Palleroni et al. 2004), while the latter, which is a very to close relative to *Chromohalobacter israelensis*, can catabolize aromatic compounds in environments with high salinity (García et al. 2005). The moderately halophilic bacterium *Chromohalobacter salexigens* and *Halomonas ventosae* (Arahal et al. 2001, Martínez-Cánovas et al. 2004) were also identified (1.1 % each of oil sludge 16S clone library). *Idiomarina loihiensis* (4.4 % of oil sludge 16S Clone Library) is another halophilic bacterium which was originally isolated from hydrothermal vents on the Loihi Seamount, Hawaii (Donachie et al. 2003). The halophilic isolates are considered to have degradative potential and play an important ecological role through specific catabolic abilities (García et al. 2005). They can be utilised for biotechnological and industrial applications via their extracellular hydrolytic enzymes (Martín et al. 2003).

Eighty seven clones were analyzed from the oil sample. A total of 60 % of the bacterial community that was isolated from the oil sample was found to be closely related to *E. aquimarinus* species (LMG 16607 and SS1788), which are usually found in sea water (Svec et al. 2005). Previously, these isolates were detected in a seawater sample from the Zhoushan Archipelago, China (Svec et al. 2005). Organisms closely matched to *Clostridium* species (∼99 % match) comprised a significant portion of the rest of the clone library; *Clostridium* sp. enrichment culture clone MB3_7 (19.6 % of the library) and uncultured *Clostridium* sp. clone De155 (11.6 % of the library). The *Clostridium*. sp. enrichment culture clone MB3_7 was isolated from industrially contaminated sediments of the creek Spittelwasser, Germany. It is capable of carrying out the reductive dechlorination of 1,2,3-trichlorobenzene to 1,3-dichlorobenzene (Bunge et al. 2008). Uncultured *Clostridium*. sp. clone De155 was isolated from the leachate sediment of an aged landfill in the south of Dongyang, Zhejiang Province, Chin (Liu et al. 2011). Another isolate closely matched to *Clostridium* species (∼99 % match) but present as a minor constituent of the clone library (1.1 % each) is *Clostridium* sp. CYP7, which was previously found to be amongst bacterial strains that demonstrated sulphur reduction in sulphur-rich peat soils (Yáñez 2006). In general, *Clostridium* species are responsible for incomplete degradation of a variety of organic compounds for which the end products are organic acids, CO_2_ and hydrogen by fermentation. Therefore, addition of nutrients should aid bioremediation of crude oil contaminated sites by stimulating these bacteria present in oil samples under anaerobic conditions. Another promising isolate that has a role in the bioprocess application is *Serratia fonticola*. This comprised 1.1 % of the clone library from the oil sample. *S. fonticola* is well known for its ability to carry out Fe(III) bioreduction in soluble conditions (García-Balboa et al. 2010).

## Conclusion

Novel and promising indigenous bacteria were detected in the oil sludge and oil of the heavy crude oil type produced by ARAMCO. The dominant bacterial genus identified were *Flavobacteria* and *Bacillus* in the sludge and oil samples, respectively. The most dominant bacteria in the sludge sampling site were most closely related to the novel *Flavobacteria*; uncultured *Bacteroidetes* bacterium AK.HH. More detailed investigation needs to be carried out in order to identify its growth and nutritional requirements; however, its potential is demonstrated through reduction of Fe (III). Other abundant bacteria within the sludge sample were members of the *Gammaproteobacteria* and *Spingobacteria*. The *Marinobacter* species isolated could be exploited for bioremediation due to their activity in hypersaline conditions. In addition, *H. effuse* and *Clostridium*. sp. H24.1, are the best candidates for catabolizing aromatic hydrocarbon/aromatic degradation.

The most dominant bacteria present in the crude oil sample has the closest match with the genus *Bacillus*; specifically *E. aquimarinus*. Other abundant bacteria within the sludge sample were members of the *Gammaproteobacteria* and *Spingobacteria*, whilst the oil sample also contained a significant population of *Clostridia*, including two promising isolates: *S. fonticola* and *Clostridium*. sp. CYP7. *S. fonticola* can perform Fe(III) bioreduction in soluble conditions while *Clostridium*. sp. CYP7 is known to carry out sulphate reduction coupled to lactate. These indigenous microbes can be utilized more effectively for oil bioremediation by a process of biostimulation. Further microbial ecology studies are needed to gain a better understanding of the microbial communities present and their involvement in the process of microbially influenced corrosion. This information is key to developing improved methods of monitoring, controlling microbially influenced corrosion and in developing applications for the bioremediation of oil contaminated sites.
